# Quantification of Bilateral Coronal Synostosis: Anterior Brachycephaly

**DOI:** 10.1177/1055665620982777

**Published:** 2020-12-30

**Authors:** Otto D. M. Kronig, Sophia A. J. Kronig, Léon N. A. Van Adrichem

**Affiliations:** 1 Department of Plastic and Reconstructive Surgery, University Medical Center Utrecht, Utrecht, the Netherlands

**Keywords:** quantification, cranial suture, synostosis, severity, brachycephaly

## Abstract

**Objectives:**

Very few studies focus on the quantification of severity of synostotic anterior brachycephaly. Aim of this study is to implement Utrecht Cranial Shape Quantifier (UCSQ) in brachycephaly patients to objectively quantify severity for both clinical and research purposes.

**Design:**

Retrospective study.

**Setting:**

Primary craniofacial center.

**Patients and Participants:**

Fifteen preoperative patients with bilateral coronal craniosynostosis (age <1.5 years).

**Intervention:**

Utrecht Cranial Shape Quantifier was used to quantify severity using the variables: width of frontal peak ratio, difference forehead peak and occiput peak, and width between sides of the head.

**Main Outcome Measure(s):**

The UCSQ variables were combined and related to Argenta clinical classification and cephalic index (CI) using 1-way analysis of variance (ANOVA). All parameters were derived from computed tomography scans.

**Results:**

Statistically significant differences were found between group means of UCSQ in the 3 categories of Argenta (ANOVA; *F*(2,12) = 22.461; *P* < .01). Tukey post hoc test showed a significant difference between Argenta types 1 and 2, types 1 and 3, and types 2 and 3 (all *P* < .01). Statistically significant differences were found between traditional CI and Argenta types (*F*(2,12) = 4.956; *P* = .03). Tukey post hoc test showed significantly difference between Argenta type 1 and 3 (*P* = .02). No differences were found between other types. Low correlation was found between UCSQ and CI (*r* = 0.47).

**Conclusions:**

Utrecht Cranial Shape Quantifier objectively captures and quantifies the shape of synostotic brachycephaly, and we therefore developed a suitable method to put severity of synostotic (anterior) brachycephaly into numbers.

## Introduction

Anterior brachycephaly (bilateral coronal synostosis) is the result of premature fusion of the bilateral coronal sutures and includes typical clinical features such as; a sagittally short and transversely wide skull shape, symmetrical occipital flattening, and elevation of the height of the forehead (Lajeunie et al., 1995). These features can be explained by Virchow’s law, which states that the bicoronal synostosis limits the growth in the forward and backward direction, resulting in occipital flattening and anteroposterior shortening of the skull. Compensatory growth occurs due to the open sagittal suture sideways and due to the open lambdoid sutures upward, resulting in parietal widening and increased forehead height (Virchow, 1851). Additionally, a frequently found feature is midface hypoplasia. This hypoplasia is used for differentiation between the “syndromic” and “nonsyndromic” type and is present in case of a syndromic type (Lajeunie et al., 1995). Each of the syndromic individual has a clearly different clinical appearance; however, they have the synostosis of the bilateral coronal suture in common and therefore a comparable, typical skull shape.

Currently, there is no golden standard for diagnosing and classifying anterior brachycephaly and the diagnosis is clinically established and computed tomography (CT) confirmed. Very few studies focus on the quantification of synostotic (anterior) brachycephaly (Hutchison et al., 2005; Feijen et al., 2012). However, the cephalic index (CI) can be used for determining the skull shape, and the CI is increased in case of brachycephaly (CI > 85%; Loveday & de Chalain, 2001).

A recently developed method for the classification and quantification of craniosynostoses is Utrecht Cranial Shape Quantifier (UCSQ) (Kronig et al., 2020). Utrecht Cranial Shape Quantifier is an outline-based method and has the advantage of capturing the actual skull shape variation with every 3-dimensional (3D) diagnostic system capturing the surface of the head. External landmarks (soft tissue landmarks, visible with the bare eye) are used to extract an outline of the skull shape in this study using CT scans, resulting in sinusoid curves. Specific and characteristic curves and parameters for anterior brachycephaly are found.

Therefore, the aim of this study is to implement UCSQ in anterior brachycephaly patients and quantify its severity.

## Material and Methods

### Patients

We included children with CT-confirmed bilateral coronal synostosis (age <1.5 years). The included patients were diagnosed at the Erasmus Medical Center, Sophia Children’s Hospital Rotterdam. A full head preoperative CT scan needed to be available.

The study was approved by the local medical ethics review committee (MEC-2016-467). The study was deemed a retrospective clinical study and did not require formal research ethics approval under the Medical Research Involving Human Subjects Act.

### Our Proposed Method (UCSQ)

**Figure 1.**
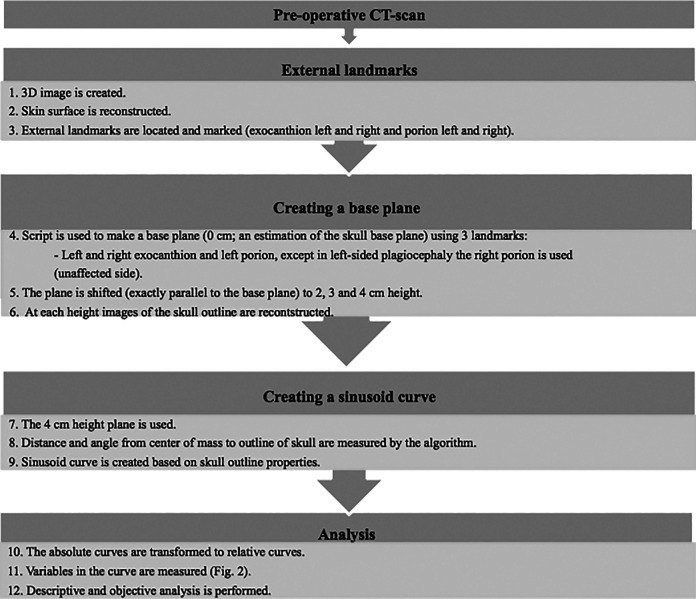
Summary of methods.

**Figure 2.**
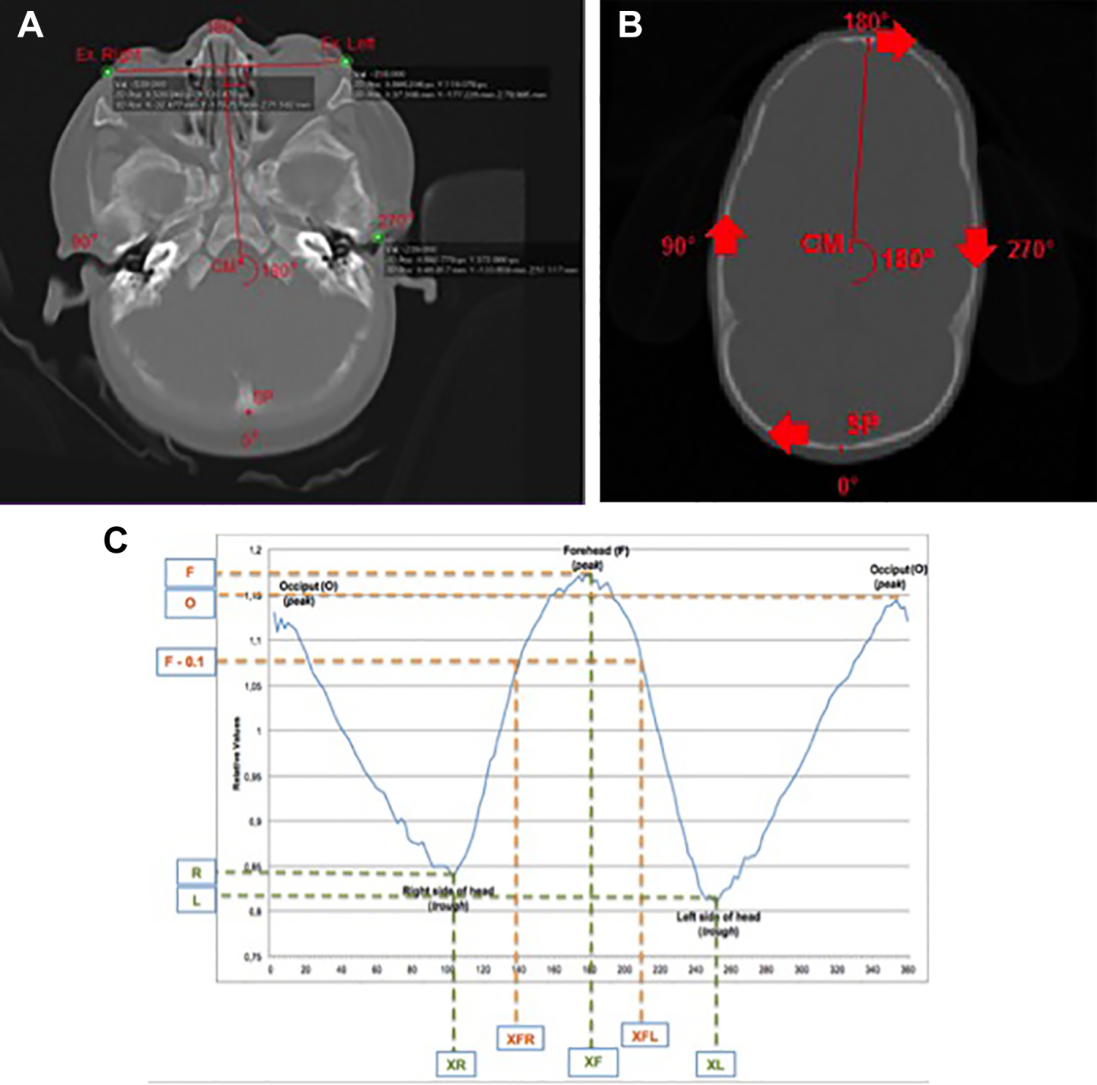
Visualization of the starting point of the curve and the resulting sinusoid curves and the used variables; the outline was made with the slices shown in (B). A, Plane on 0-cm height; this figure shows how the starting point of the curve is determined. Also, the degrees of the circle/outline are added, which are represented in the curve. B, Plane on 4-cm height; this figure shows the starting point and the direction of the curve. C, Shows the resulting curve; the different variables are marked. Curve starts at occiput (SP; B) and follows the skull outline (on CT scan) clockwise; therefore, first trough represents the right side of the head. Second peak is forehead; second trough is left side of the head. Curve stops where it started (at the occiput). CM, center of mass; F, maximum of forehead; L, minimum value of left side of head; O, maximum of occiput; R, minimum value of right side of head; SP, starting point; XF, X-value of maximum forehead value; XFL, X-value for the maximum forehead minus 0.1 on the left side; XFR, X-value for the maximum forehead minus 0.1 on the right side; XL, X-value of the minimum value of the width on the left side; XR, X-value of the minimum value of the width on the right side. CT indicates computed tomography.

The methodology for the quantification of craniosynostosis developed in our previous study (UCSQ) is used (Figure 1) (Kronig et al., 2020). The curves, generated by the UCSQ method, were analyzed for different variables (Table 1 and Figure 2). Figure 2 shows how the curves are derived and an example of an obtained curve. The curve starts at the occiput and skull outline is followed clockwise. After the first peak, resembling the occiput, the curve decreases, because the distance from the center of mass to the right side of the head is shorter than the distance from center of mass to the forehead or occiput. The second peak resembles the forehead; again, the curve decreases to the left side of the head and increases to the occiput.

**Table 1.** Extracted and Calculated Variables From Curve.

Extracted variable	Abbreviation	Extracted variable	Abbreviation
Maximum value of forehead peak	F	Maximum value of occiput peak	O
X-value (in degrees) for maximum forehead minus 0.1 (F-0.1) on left side	XFL	X-value (in degrees) for maximum forehead minus 0.1 (F-0.1) on right side	XFR
Minimum value of left side of head (trough)	L	Minimum value of right side of head (trough)	R
X-value (in degrees) of minimum value of width on left side	XL	X-value (in degrees) of minimum value of width on right side	XR
Calculated variable	Formula	Calculated variable	Formula
Width of frontal peak ratio	(XFL-XFR)/(F-0.1)	Difference forehead peak and occiput peak	F-O
Width between both sides of the head	XL-XR	CI UCSQ	(R + L)/(F + O)

Abbreviations: UCSQ, Utrecht Cranial Shape Quantifier.

Variables used for quantification of severity of brachycephaly are the following: width of frontal peak ratio, difference forehead peak and occiput peak (F-O) and width between both sides of the head (XL-XR). Included patients were ranked separately according to these variables, in which lowest width of peak ratio was given the lowest rank number (1), the lowest XL-XR was given the lowest rank number (1), and the highest F-O was given the lowest rank number (1). All ranking numbers were added.

Additionally, in order to differentiate between the different levels of severity of brachycephaly, we used the most distinctive variables for brachycephaly, namely the aforementioned: width of frontal peak ratio, difference forehead peak and occiput peak, and width between both sides of the head. As stated before, the brachycephalic skull is sagittally shorter and transversely wider compared to the normal skull. Therefore, the difference between the mean values of a control skull for the previous variables and those of a brachycephaly patient is indicative for severity. We used the mean values from the control patients, as reported in our previous study (Kronig et al., 2020).

The following calculation to determine cutoff values and the different classes of severity (mild, moderate, and severe) was developed: (Difference forehead peak − occiput peak − 0.004) × −4000 + (Width between both sides of the head − 164.4) × 3 + (Width of frontal peak ratio − 82.08) × 1.3.

In this calculation, the values 0.004, 164.4, and 82.08 are the values of the variables (difference forehead peak and occiput peak, width between both sides of the head, and width of frontal peak ratio respectively) in control patients. In the calculation, the differences between the variables in brachycephaly patients and control patients are multiplied (by −4000, 3, and 1.3) in order to give each variable the same weight in the resulting outcome. Following, cutoff values for each subgroup of severity were proposed.

### Argenta Classification

All patients were graded by 3 experienced raters (1 craniofacial plastic surgeon and 2 trained students) according to the Argenta classification method using 3D-CT scan (Table 2) (Argenta et al., 2004). Mean Argenta classification was calculated for each patient and used to quantify the severity.

**Table 2.** Clinical Classification After Argenta.

Argenta	Description
Type 1	Depression of the central skull at the confluence of the lambdoids with the sagittal suture. Position of the ears, forehead, and face is otherwise normal.
Type 2	Type 1 + widening of the skull in its posterior half as the brain attempts to decompress.
Type 3	Type 2 + vertical growth of the posterior skull and/or temporal bulging. This is a reflection of an extremely flat, constricted skull.

### Cephalic Index

Traditional CI represents the ratio of maximum cranial width to maximum cranial length multiplied by 100 (CI = biparietal diameter (BPD)/occipitofrontal diameter (OFD) × 100). The CI gives information of the head shape and the severity of the malformation of the skull. A CI >85% is considered brachycephaly, CI of 75% to 85% plagiocephaly or normocephaly and <75% scaphocephaly (Loveday & de Chalain, 2001).

Cephalic index can also be measured using the curve following UCSQ. The maximum cranial width in the new method was measured by multiplying the mean value of both sides of the head by 2 (BPD) and the maximum cranial length (OFD) was measured by adding the value of the maximum of forehead to the maximum value of the occiput.

### Statistical Analysis

Level of agreement between observers was assessed for Argenta classification by calculating intraclass correlation coefficients (ICC). The ability of a test to show interobserver reliability was evaluated using the 2-way random effects model assuming an average measurement and absolute agreement. An ICC of 1 means perfect reliability, and an ICC of 0 shows poor reliability. The outcomes of the ICC are characterized as poor (0.00-0.20), fair (0.21-0.40), moderate (0.41-0.60), good (0.61-0.80), or excellent (0.81-1.00) (Landis & Koch, 1977).

One-way analysis of variance (ANOVA) or Kruskal-Wallis test was used to compare UCSQ (added rank numbers of variables of UCSQ) and Argenta classification (both traditional and UCSQ), and CI and Argenta classification. The used test was based on normality of data and appropriate post hoc tests were used (Tukey post hoc test).

Pearson correlation coefficient or Spearman rank correlation coefficient was used to determine correlation between UCSQ and CI, and between the traditional CI and the CI of UCSQ, depending on normality. The accepted guidelines for interpreting the correlation coefficients are: +1 indicates a perfect positive linear relationship, −1 indicates a perfect negative linear, and 0 indicates no linear relationship (Ratner, 2009). The size of a correlation coefficient can be interpreted as follows: negligible correlation (0.00-0.30), low (0.30-0.50), moderate (0.50-0.70), high (0.70-0.90), and very high (0.90-1.00) (Hinkle et al., 2003).

Statistical analyses were performed using the Statistical Package for the Social Sciences (SPSS) for Windows (Version 21, SPSS Inc). Statistical significance was set at a *P* value less than or equal to .05.

## Results

### Patient Characteristics

We included 15 children with anterior brachycephaly. Mean age at preoperative CT scan was 5.5 (1-18) months. There were 6 male and 9 female patients (40% vs 60%, respectively).

### Intraclass Correlation Coefficient

Intraclass correlation coefficient for Argenta classification was calculated. The level of agreement between the 3 observers was 0.84, showing excellent reliability.

### Utrecht Cranial Shape Quantifier

**Figure 3.**
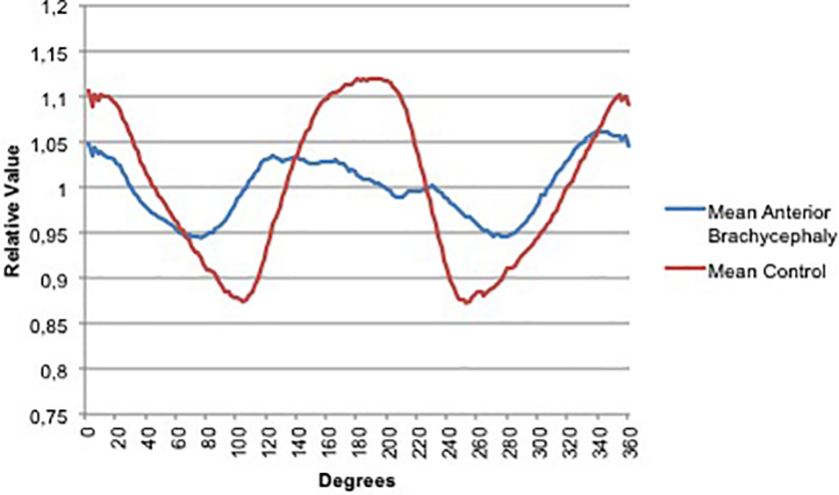
Mean curves for anterior brachycephaly (n = 15) and control patients (n = 5).

Figure 3 shows the mean curves for anterior brachycephaly (n = 15) compared to control (n = 5) patients. Control patients were as included in our previous study (Kronig et al., 2020). Used variables for quantification according to UCSQ were width of frontal peak ratio (mean 158.6 [69.3-232.2]), difference between peak of forehead and peak of occiput (mean 0.03 [0.01-0.10]), and width between both sides of the head (mean 199 [118-242]).

Mean of the calculation for severity of brachycephaly ((Difference forehead peak − occiput peak − 0.004) × −4000 + (Width between both sides of the head − 164.4) × 3 + (Width of frontal peak ratio − 82.08) × 1.3)) was 302.52 (−53.71 to 591.21). Mean (Difference forehead peak − occiput peak − 0.004) was −0.02 (−0.01 to 0.04), mean (Width between both sides of the head − 164.4) was 34.80 (−46.40 to 77.60), and mean (Width of frontal peak ratio − 82.08) was 76.50 (−12.75 to 150.16).

**Figure 4.**
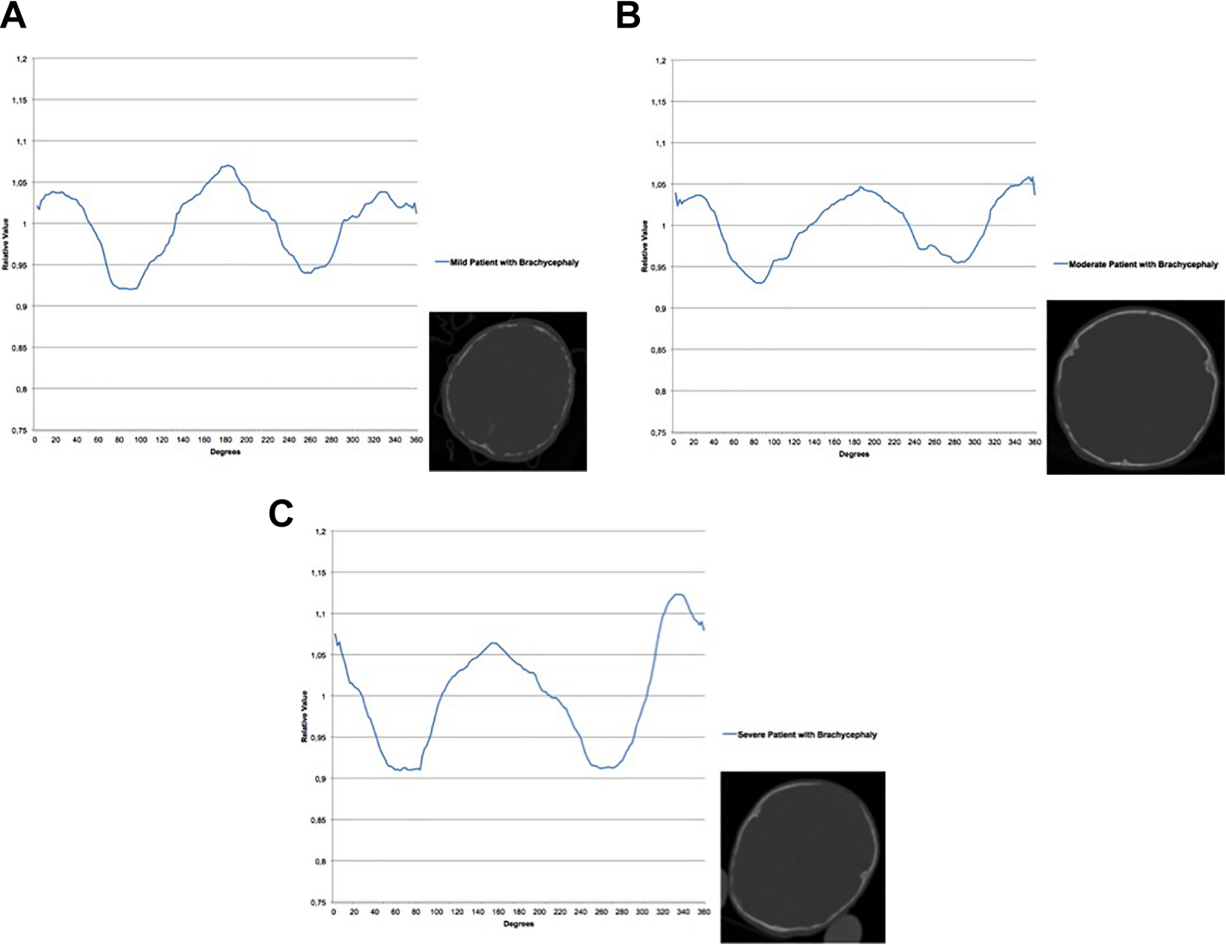
Curves and skull slices of 3 patients of the different classes of severity according to our UCSQ severity classification. A, Patient with mild brachycephaly. B, Patient with moderate brachycephaly. C, patient with severe brachycephaly. UCSQ indicates Utrecht Cranial Shape Quantifier.

Based on our calculation of severity of brachycephaly ((Difference forehead peak − occiput peak − 0.004) × −4000 + (Width between both sides of the head − 164.4) × 3 + (Width of frontal peak ratio − 82.08) × 1.3), we propose the following cutoff values in order to classify severity: mild < 200, moderate 200 – 350, severe > 350. Figure 4 shows examples of 3 patients of the different classes of severity; these photographic representations visualize how the sinusoid curves correlate to the skull shape.

### Other Variables

Mean Argenta classification was 2 (type 1: 3; type 2: 8; type 3: 4). Mean traditional CI was 0.96 (0.83-1.12). Mean CI of UCSQ was 0.87 (0.79-0.94).

### Comparison UCSQ and Existing Methods

Statistically significant differences were found between group means of UCSQ in the different categories of Argenta classification by using 1-way ANOVA (*F*(2,12) = 22.461; *P* < .01). A Tukey post hoc test showed that the mean score for Argenta type 1 was significantly different from type 2 (mean −12.69, SE 3.09, *P* < .01), between mean score of type 1 and 3 (mean −23.38, SE 3.49, *P* < .01), and a statistically significant difference between type 2 and 3 (mean −10.69, SE 2.80, *P* < .01).

Statistically significant differences were found between group means of traditional CI and Argenta classification by using 1-way ANOVA (*F*(2,12) = 4.956; *P* = .03). A Tukey post hoc test showed that the mean score for Argenta type 1 was significantly different from type 3 (mean −0.15, SE 0.05, *P* = .02). No statistically significant differences were found between the other types (types 1 and 2 mean −0.09, SE 0.04, *P* > .05 and between type 2 and 3 mean −0.05, SE 0.04, *P* > .05).

No statistically significant differences were found between group means of CI by UCSQ and Argenta classification by using 1-way ANOVA (*F*(2,12) = 2.647; *P* > .05). Low correlation was found between UCSQ and CI (*r* = 0.47), and moderate correlation was found between traditional CI and calculated CI of UCSQ (0.55).

## Discussion

Very few studies focus on the quantification of severity of synostotic (anterior) brachycephaly. However, for research purposes, as well as patient education and clinical outcome, it is important to be able to quantify severity of a given diagnosis. The present study tried to solve this problem.

In general, brachycephaly is less discussed in the literature, probably because of the posterior, symmetric skull deformity without ear shift and facial scoliosis (Siegenthaler, 2015). However, one known classification type is the Argenta classification system, which is a visual assessment of the skull deformity (Argenta et al., 2004). The patients are clinically examined in 4 positions: from the front, above, back, and side. However, the Argenta classification is a subjective method because the classification is not quantitative and has therefore less scientific power. When comparing UCSQ to Argenta classification, we found significant difference between all mean scores of Argenta subtypes. This indicates that UCSQ quantifies the described visual deformities of the Argenta classification. When comparing traditional CI to Argenta classification, only differences between types 1 and 3 of Argenta classification can be found.

Cephalic index is a well-known and used method in clinical practice; however, CI is indicative for a certain diagnosis, and it does not correlate to severity, as found in our study (moderate correlation). Cephalic index only takes the longest skull width and length into account and does not capture the skull shape. Additionally, there is no consistent cutoff point in the literature defining brachycephaly. It has been defined as a CI of ≥80% (Bass, 1987; Hall et al., 1989), ≥82% (Dekaban, 1977), and ≥85% (Loveday & de Chalain, 2001).

In contrast, UCSQ captures the skull shape of brachycephaly patients (and patients of each type of craniosynostosis). The resulting curves show a specific pattern, which are relatable to the visual deformities of brachycephaly. The most distinctive variables of brachycephaly are found to be width of frontal peak ratio (broad peak is specific for brachycephaly) representing the width of the forehead, difference between peak of forehead and peak of occiput (due to fusion of both the coronal sutures a circular head shape develops, the more severe the brachycephaly, the rounder the head, resulting in a difference less than 0), and width between both sides of the head (the larger this distance, the more severe the brachycephaly).

Based on our calculation of severity of brachycephaly ((Difference forehead peak − occiput peak − 0.004) × −4000 + (Width between both sides of the head − 164.4) × 3 + (Width of frontal peak ratio − 82.08) × 1.3), we propose the following cutoff values in order to classify severity: mild < 200, moderate 200 to 350, severe >350. However, these cutoff values are only based on 15 patients, and further validation is needed in future research.

In the clinical setting, CI is easy to obtain and gives valuable information on parietal width to fronto-occipital length. However, to be better informed on the width of the forehead and the relation of the frontal length to the occipital length, UCSQ is superior. Therefore, UCSQ is a promising tool for outcome studies and scientific analysis.

Several limitations should be considered when interpreting the results. First, this study would include the general drawback of any retrospective study. Secondly, we used data from only one craniofacial center, resulting in an apparent relatively small patient group. However, we included a homogeneous group of patients, with regard to age and preoperative status. Due to the rarity of synostotic brachycephaly, the included group of patients is substantial and large enough for statistical significant results.

In conclusion, using UCSQ, we are able to objectively capture and quantify the skull shape of brachycephaly patients, and therefore, we developed a suitable method to quantify the severity of brachycephaly. In our craniofacial unit, the diagnosis bilateral coronal synostosis was made by CT scanning. A more specific diagnosis was made on clinical (facial) features and genetic testing. However, due to the lack of an objective method for establishing severity, we started the present study. The application of UCSQ will lead to accurate classification of the severity of brachycephaly. Future research can focus on the application of this method on 3D-photogrammetry, which is less invasive and not damaging (no radiation load, no need for sedation) for children. When 3D photogrammetry is used to perform UCSQ analysis, it can be used for monitoring skull shape and growth. Furthermore, UCSQ may be used for evaluation of (varying) surgical techniques in comparison to nonsurgical management. However, further research is necessary.
